# *Notes from the Field*: Increase in Coccidioidomycosis — Arizona, October 2017–March 2018

**DOI:** 10.15585/mmwr.mm6744a6

**Published:** 2018-11-09

**Authors:** Carla P. Bezold, Mohammed A. Khan, Guillermo Adame, Shane Brady, Rebecca Sunenshine, Ken Komatsu

**Affiliations:** ^1^Epidemic Intelligence Service, CDC; ^2^Arizona Department of Health Services, Phoenix, Arizona; ^3^Maricopa County Department of Public Health, Phoenix, Arizona; ^4^Department of Epidemiology, Rollins School of Public Health, Emory University, Atlanta, Georgia; ^5^Career Epidemiology Field Officer Program, CDC.

Beginning in October 2017, the Arizona Department of Health Services (ADHS) noted an increase in the number of reported cases of coccidioidomycosis (Figure). According to provisional data (not finalized), the incidence in December 2017 (17.2 per 100,000 population) represented the highest monthly rate in the last 5 years, surpassing the previous peak of 14.2 cases per 100,000 population in September 2015. In total, 4,827 cases of coccidioidomycosis were reported to ADHS during October 2017–March 2018. Whereas case counts typically increase during these months, this particular period represented a 58.3% increase over the 3,050 cases reported during the same months the previous year and a 50.3% increase over the 6-month average of 3,211 cases reported during October–March for the years 2013–2017.

**FIGURE:**
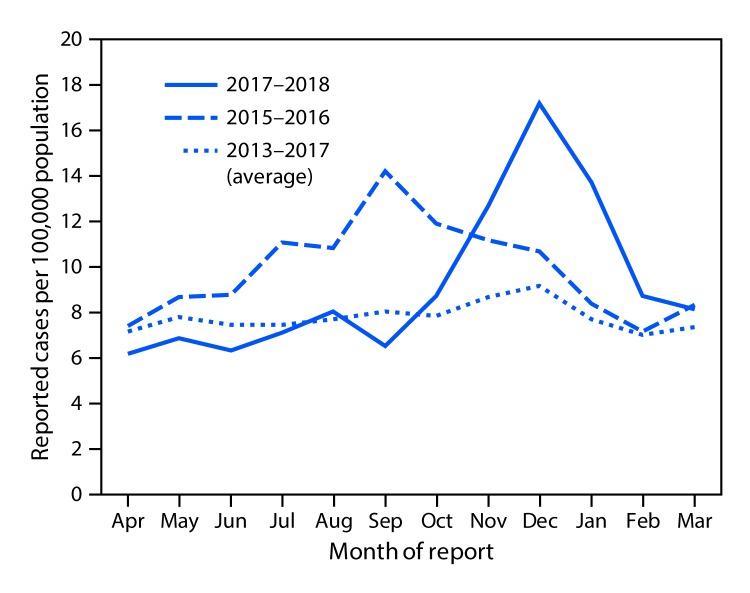
Monthly incidence of coccidioidomycosis — Arizona, April 2013–March 2018

Coccidioidomycosis (Valley fever) is an infectious disease caused by inhalation of *Coccidioides* spores; approximately 40% of infected persons experience signs and symptoms including fever, cough, fatigue, chest pain, shortness of breath, and rash. *Coccidioides* is endemic in soil in the southwestern United States (*1*). The majority of reported U.S. coccidioidomycosis cases occur in Arizona (*2*), and incidence is seasonal: the highest number of reported cases in Arizona typically occurs during the fall and winter months.* Because of the high number of cases in Arizona and the high predictive value of a positive laboratory result, Arizona’s coccidioidomycosis case definition requires only laboratory evidence to confirm a case (*3*). Laboratory evidence can include detection of anticoccidioidal immunoglobulin M (IgM) or immunoglobulin G (IgG) antibodies; culture, histopathologic, or molecular evidence of *Coccidioides* spp.; or coccidioidal skin test conversion after illness onset.

During October 2017–March 2018, the median age of persons with reported coccidioidomycosis was 56 years (interquartile range [IQR] = 39–69 years); approximately half (50.5%) of patients were male. Age and sex distributions were similar to those observed during October 2016–March 2017, with a median age of 57 years, (IQR = 40–69); 51.2% of patients were male. Approximately 90% of persons with reported coccidioidomycosis in Arizona reside in the three most populous counties (Maricopa, Pima, and Pinal). During October 2017–March 2018, 3,674 cases were reported in Maricopa County (87.0 cases per 100,000 population), a 70.5% increase over the 2,157 cases (52.0 per 100,000 population) reported during the same period the preceding year. The number of reported cases and incidence also increased, but less sharply, in Pima County (31.5% increase, 601 cases, 58.6 per 100,000 population versus 457 cases, 45.1 per 100,000 population the preceding year) and Pinal County (29.5% increase, 329 cases, 76.9 per 100,000 population versus 254 cases, 61.5 per 100,000 population).

To evaluate the possibility of laboratory or reporting artifact, data were reviewed to assess the proportion of cases that were coccidioidomycosis-positive by enzyme immunoassay (EIA) for IgM antibodies alone. EIA IgM alone has been reported to have lower specificity in some circumstances compared with other testing methods (*4*). There were 4,638 cases reported during October 2017–March 2018 where the type of laboratory test used could be classified; 602 (13.0%) tested positive by EIA IgM alone, compared with 316 of 2973 (10.6%) during the same months 1 year before. This slightly higher proportion of cases testing positive by EIA IgM alone is insufficient to explain the magnitude of the increase in cases during October 2017–March 2018. No known changes in provider or laboratory reporting occurred during this time.

Reasons for the current increase in reported coccidioidomycosis are unknown but might include weather and environmental factors, including precipitation, which can facilitate growth of *Coccidioides*, followed by high temperatures and drought, which can facilitate distribution (*5*). Preliminary data suggest that 2017 was uncharacteristically warm and dry in central Arizona.^†^ In addition, during 2016–2017, Maricopa County experienced the largest population gain of any county in the United States.^§^ An increase in the number of susceptible persons and dust disturbance, resulting from increased residential construction, might have contributed to the increased incidence of coccidioidomycosis. Further investigation of the causes of increased coccidioidomycosis in areas with endemic transmission is crucial to informing strategies to prevent disease and educate providers and the public regarding the importance of appropriate diagnosis and management of coccidioidomycosis.

## Footnotes

*
https://www.azdhs.gov/documents/preparedness/epidemiology-disease-control/valley-fever/reports/valley-fever-2016.pdf .

† https://www.weather.gov/psr/Year_in_Review_2017.

§
https://www.census.gov/newsroom/press-releases/2018/popest-metro-county.html .
